# Behavioral Therapy and Fluoxetine Treatment in Aggressive Dogs: A Case Study

**DOI:** 10.3390/ani10050832

**Published:** 2020-05-11

**Authors:** Rosangela Odore, Diego Rendini, Paola Badino, Giulia Gardini, Giulia Cagnotti, Valentina Meucci, Luigi Intorre, Claudio Bellino, Antonio D’Angelo

**Affiliations:** 1 Department of Veterinary Sciences, University of Turin, Largo Braccini 2, 10095 Grugliasco (TO), Italy; rosangela.odore@unito.it (R.O.); doc.rendini@gmail.com (D.R.); giulia.gardini@unito.it (G.G.); giulia.cagnotti@unito.it (G.C.); claudio.bellino@unito.it (C.B.); antonio.dangelo@unito.it (A.D.); 2 Department of Veterinary Sciences, University of Pisa, Viale delle Piagge 2, 56124 Pisa, Italy; valentina.meucci@unipi.it (V.M.); luigi.intorre@unipi.it (L.I.)

**Keywords:** dog, aggression, serotonin, fluoxetine, norfluoxetine

## Abstract

**Simple Summary:**

Despite dog aggression representing a frequent and serious threat to public health, currently there are no licensed drugs for treating dog aggression. Treatment approaches include behavior management programs and empiric administration of fluoxetine for extended periods. The drug is used at 1–2 mg/kg of body weight every 24 h according to the clinician’s prescription. Studies concerning long-term dosage schedules and the effects of fluoxetine on clinical and blood parameters have not been undertaken in veterinary medicine. In the present study, fluoxetine (1.5 mg/kg/die PO) combined with a behavior modification program were used for treatment in eight dogs with a diagnosis of dominance-related aggression. Clinical outcomes for fluoxetine, norfluoxetine, and serotonin circulating levels were periodically evaluated over a six-month period. After one month of treatment, significant clinical improvement was observed, although dogs were classified as fully responsive starting from T2 (two months of treatment). At the end of the follow-up (six months of treatment), a correlation between norfluoxetine levels and clinical scores (*r* = 0.75, *p* < 0.05) was observed. Blood serotonin levels were significantly decreased. The results suggest that the dosage schedule is useful in the management of dominance aggression in dogs and that norfluoxetine levels seem reliable in predicting clinical efficacy.

**Abstract:**

Canine aggression is a major concern, affecting millions of people worldwide, and treatment can be challenging even for skilled veterinarians. Empiric use of fluoxetine is sometimes attempted, although few data regarding long-term effects in aggressive dogs are available. The aim of the study was to investigate clinical effectiveness of fluoxetine (1.5 mg/kg/die PO) combined with a behavior modification program for treatment of canine dominance-related aggression. Circulating levels of fluoxetine, norfluoxetine, and serotonin (5-HT) were also measured. Eight dogs with a diagnosis of dominance aggression (owner-directed) were enrolled. Before treatment (T0), and after one (T1), two (T2), four (T3), and six (T4) months of fluoxetine administration, clinical outcomes were graded using a five-point frequency scale (0–4), and blood samples were collected to measure fluoxetine/norfluoxetine (high-performance liquid chromatography) and 5-HT (ELISA) levels. Following treatment, a decrease in behavioral test scores was observed at T1–T4. Increasing concentrations of circulating fluoxetine and norfluoxetine were measured throughout the follow-up. Correlation between norfluoxetine levels and clinical scores was observed at T4. Starting from T1, a significant decrease in 5-HT levels was observed. Our data suggest that fluoxetine (1.5 mg/kg/day) when associated with behavior treatment is effective in controlling canine aggression over a six-month period, and that, in dogs norfluoxetine levels seem reliable in predicting clinical efficacy.

## 1. Introduction

Canine aggression toward people is one of the most common behavioral problems seen at animal behavior clinics. According to Polo et al. [1] dog bites affect 1.5% of the US population annually, whereas Belgium has an annual frequency of 22 bites per 1000 children, and about 136,000 dog bite incidents occur annually in the Netherlands. The consequence for dogs of showing aggression towards people is often euthanasia or relinquishment. However, when owners choose to attempt treatment, the typical therapeutic approach involves appropriate behavior modification exercises coupled with an adjunctive pharmacologic support where indicated. Identifying the motivation of the aggression is the first step in determining how to treat the dog. Dominance aggression, although rare, is an offensive form of aggression seen during competitive interactions over the control of resources and/or in response to a perception of challenge to the animal’s social status. It usually develops at social maturity (18–24 months of age) [2]. The goal of treatment is to modify the dog’s behavior and to manage their environment to prevent aggressive threats. In some cases, drug therapy is a helpful adjunctive therapy for this kind of behavioral problem [3]. However, although dog aggression toward owners represents a frequent and serious threat to public health, currently there are no licensed drugs for treating aggression.

Empiric treatment is primarily focused on serotonin (5-HT). Several studies in humans and laboratory animals have, in fact, documented that the 5-HT system is associated with behavioral inhibition [4]. In dogs, lower levels of the 5-HT metabolite 5-hydroxyindolacetic acid (5-HIAA) have been observed in the cerebrospinal fluid of dominant, aggressive subjects than in nonaggressive ones, as well as modifications in serum 5-HT levels [5,6]. Moreover, it has been suggested that modifications of 5-HT receptor densities and of the function in various brain regions of aggressive dogs do occur [7]. These observations have led to the therapeutic use of selective serotonin reuptake inhibitors (SSRIs) with the aim to manipulate 5-HT concentrations in the synaptic cleft of aggressive dogs [8]. Among SSRIs, fluoxetine, approved for use in dogs for separation anxiety (Reconcile^®^, Eli Lilly, Indianapolis, Indiana), is the drug with the longest history of use for behavior problems in dogs, including aggression. In such cases, the drug is used at 1–2 mg/kg of body weight every 24 h, according to the clinician’s prescription [9]. Similarly to all other SSRIs, fluoxetine requires continuous prolonged administration to produce therapeutic changes. Few data regarding fluoxetine’s effects in aggressive dogs are available. In a study by Dodman and colleagues [10], the drug induced a significant reduction in owner-directed aggression after 3 weeks of treatment at 1 mg/kg. Moreover, according to Rosado et al. [11], a 30-day fluoxetine treatment causes a decrease in peripheral 5-HT concentration, whereas circulating cortisol levels were unaffected. Therefore, further scientific support is needed to facilitate clinicians in the diagnostic *iter* and in the choice of the appropriate treatment protocol. Key issues in performing this kind of study are the definition of inclusion criteria and the classification of aggression. The aim of the present study was to assess the behavioral effects of a six-month-long treatment in dogs affected by dominance aggression directed towards owners. Moreover, at different experimental time points during the clinical follow-up, blood fluoxetine, its principal active metabolite norfluoxetine, and 5-HT levels were measured in order to correlate the clinical findings with pharmacodynamic and pharmacokinetic effects.

## 2. Materials and Methods

### 2.1. Animals

On the basis of inclusion criteria, out of more than 108 dogs referred to the Veterinary Teaching Hospital of the University of Turin for episodes of aggression towards owners for at least 2 months and not more than 4, 8 dogs were included in the study. The group (7 males and 1 females) consisted of dogs of different breeds: German Shepherd (1), Cocker Spaniel (1), Jack Russell (1), Boxer (1), and mixed breed (4). The mean age was 3.6 years (ranging from 1.2 to 6 years). The dogs showed no clinical signs but aggression and had received no pharmacological treatment. At the time of enrollment, the presence of any concurrent medical condition contributing to aggression was excluded by means of physical and neurological examination carried out by a board-certified neurologist and by serum biochemistry, complete blood count, and thyroid hormone levels (TSH andtotal thyroxine). Among inclusion criteria, there was the possibility to manage dogs without using sedation.

The diagnosis of dominance-related aggression (owner-directed) was made by a behaviorist expert on the basis of anamnesis and clinical evaluation. The behavioral case history was collected by a questionnaire filled out directly by the owner, who was asked to describe a series of situations (e.g., food-related aggression; disturbed while resting; physical contact; postural or behavioral provocation by the victim) in which the dog displayed aggressive behavior [10]. Clinical diagnosis of aggression was established on a five-point frequency scale (0–4; higher score indicates more severe disease) for three different items concerning: (a) frequency of aggression episodes (F), (b) aggression intensity (I), (c) distance from the aggressive reaction to when the stimulus did not appear (D) (Table 1). The distance was defined according to the concept of the proxemic bubble, personal space surrounding the body that defines a sort of protective bubble: intimate (from 0 to about 0.5 m), personal (0.5 to 1.2 m), social (1.2 to 3 m), and public (greater than 3 m).

A score ranging from 0 to 12 was assigned to each dog at the time of enrollment and at each further experimental time point. Dogs were considered for treatment if their clinical score was ≥8 at time of enrollment (T0).

During the follow-up, animals having a score ≤3 were considered to be fully responders to the therapy.

### 2.2. Treatment and Experimental Time Points

All the owners were provided with a series of recommendations concerning interactions with the dog and guidance on how to administer the drug and were asked to sign an informed consent. Fluoxetine was prescribed at 1.5 mg/kg PO, every 24 h for 6 months. Pharmacological therapy was conducted for six months, as well as the rehabilitation program adopted in this study. Considering the aggressive behavior of the dogs included and the potential risks of harm for their owners, we decided to associate both therapeutic approaches for the entire length of the study. The drug was prepared ad hoc in form of a gel capsule depending on the dog’s weight. The cognitive behavioral therapy was based on: (a) ethological management of owner-dog relationship; (b) dogs’ self-control and frustration management; (c) concentration of attention; and (d) problem-solving activity. Dogs were revaluated after a period of one (T1), two (T2), four (T3) and six (T4) months of fluoxetine treatment. Before treatment (T0), and at each further experimental time point, animals underwent physical and behavioral examination, and the clinical score was updated. At each time point, the correct execution of the exercises was verified by the same veterinary behaviorist (D.R.) by means of an interview with the owner, aimed at evaluating the owner’s ability to implement the measures required by behavior therapy and the practical execution of exercises.

Below, we briefly describe the exercises proposed, divided by each category.

Ethological Management of Owner-Dog Relationship:

- The owners were instructed to avoid conflicting situations and punishment;
- The dogs were ignored if nervous and aggressive behaviors were shown;
- The owners were instructed to improve the relationship with their dogs through correct communication and proxemics;
- The owners managed social resources;
- The owners took the dogs on more walks;
- The owners were instructed to create a safe place for their dogs at home.

Self-Control and Frustration Management:

- Obedience training was provided to teach desirable behaviors (i.e., “sit”, “lie down”, “come here”, and “stay”), which were rewarded when the dogs performed them in a calm way, with the time between exercise execution and reward gradually increased.

Centripetation of Attention:

- The dogs were taught to look at their owners’ eyes on command to get their attention or to obtain a treat.

Problem-Solving Activity

- Snuff tracks;
- Shell-scent game.

Furthermore, blood samples were collected from the cephalic vein into lithium heparin, EDTA, and serum tubes, and centrifuged at 2500 g at 4 °C for 15 min. All samples were collected in the morning between 10:00 and 12:00 a.m., before fluoxetine daily administration. Aliquots of plasma and serum were stored at −80 °C until further analysis.

Animal sampling was performed by a veterinary surgeon after receiving the consent of the owner.

### 2.3. Measurement of Plasma Fluoxetine and Norfluoxetine Concentrations

Fluoxetine and norfluoxetine plasma concentrations were determined using a high-performance liquid chromatography (HPLC) method. The extraction of plasma samples was conducted according to the method described by Vlase et al. [12], with minor modifications. Briefly, 1000 μL of plasma was alkalinized with 250 μL of 0.2 M NaOH, vortexed for 10 s and extracted with 5 mL of a solution containing hexane-isoamyl alcohol 97/3%. The samples were vortexed for 10 s, placed in a rotary samples mixer for 15 min, and centrifuged at 3500 g for 10 min. The supernatant was separated from the aqueous phase and acidified with 250 μL of 1% H_3_PO_4_, vortexed for 10 s, placed in a rotary samples mixer for 15 min, and centrifuged at 3500 g for 10 min. One hundred microliters of acidic aqueous phase was collected with a Hamilton syringe and injected into the HPLC. The HPLC system consisted of a binary gradient pump (SpectraSYSTEM^®^ P2000 Thermo Finnigan, Waltham, MA, USA) connected to a UV-VIS detector (SpectraSYSTEM^®^ 3000 Thermo Finnigan, Waltham, MA, USA) and an autosampler (SpectraSYSTEM^®^ AS3000 Thermo Finnigan, Waltham, MA, USA). The software used for data processing was Chromquest^®^ (Thermo Finnigan, Waltham, MA, USA). Separation was achieved on a C_18_ SunFire^TM^ column (4.6 × 250 mm, 5 μm particle size) (Waters, Milford, MA, USA). The detection was done at 226 nm. The mobile phase consisted of acetonitrile and 40 mM potassium dihydrogen phosphate buffer (pH adjusted at 2.3 with 85% phosphoric acid) in the ratio 31:69 (*v*/*v*). The mobile phase was delivered at a flow rate of 1 mL/min. The autosampler injection volume was 100 μL. The method was linear in the range of 20 and 1500 ng/mL for both fluoxetine and norfluoxetine. The linearity, obtained by linear regression of calibration curves in spiked plasma samples, showed values of *r*^2^ > 0.99. The intra-day and inter-day variability for both fluoxetine and norfluoxetine was <15% and <20%, respectively. For both analytes, the limit of quantification and the limit of determination were 20 and 10 ng/mL, respectively. The average extraction recovery for both fluoxetine and norfluoxetine was >90%.

Fluoxetine and norfluoxetine were purchased from Sigma-Aldrich (Milan, Italy). Solvents HPLC grade were purchased from Labscan (Hasselt, Belgium). Fluoxetine and Norfluoxetine stock solutions (1 mg/mL) prepared in acetonitrile were stored at −20 °C. Working solutions, prepared by diluting stock solutions with acetonitrile, were stored at 4 °C.

### 2.4. Serum 5-HT Determination

Serum 5-HT was measured with a commercial ELISA kit (Serotonin-ELISA; DLD Dianostika GMBH, Hamburg, Germany). Concentrations were expressed in ng/mL.

### 2.5. Statistical Analysis

Assumption of normal distribution of residuals was tested using the Shapiro–Wilk normality test. Statistical analysis was performed by means of repeated measures one-way ANOVA and Tukey’s Multiple Comparison Test (limit set at *p* < 0.05). In the modes, time was considered as an independent variable while clinical scores and fluoxetine/norfluoxetine/5-HT levels were considered as dependent variables (GraphPad Prism, Version 8; San Diego, CA, USA). To evaluate correlation between clinical scores and fluoxetine/norfluoxetine/5-HT levels, the Pearson’s correlation was used.

## 3. Results

**Figure 1:**
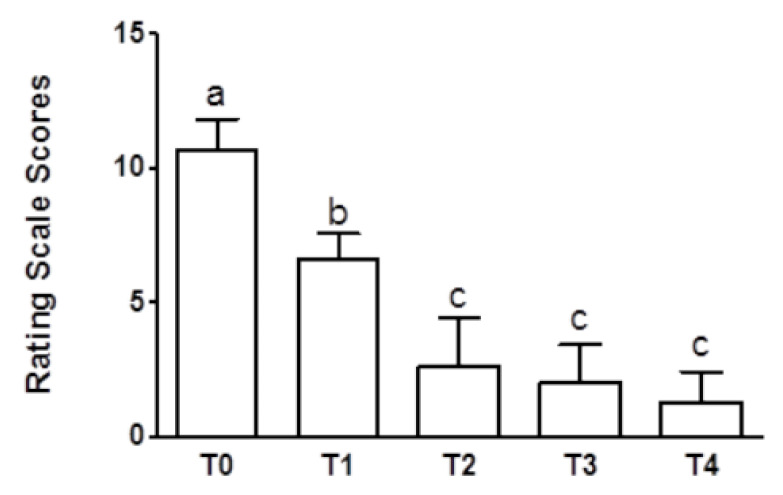
Rating scale scores (0–12) of aggressive dogs before treatment (T0), and after one (T1), two (T2), four (T3), and six (T4) months of fluoxetine treatment (T0 vs. T1 vs. T2-T4 = *p* < 0.001, *n* = 8). a, b, c: statistically significant differences are present among the values reported in the bar of the graph.

All the dogs were clinically healthy and biochemical parameters were within the expected ranges at time of admission (data not shown). Following treatment, all the owners reported to have noticed an improvement of the condition, which was reflected in the significant decrease of the behavioral test score observed at T1–T4 (Figure 1). A statistically significant (*p* < 0.001) improvement between T0 and T1 was observed. However, dogs were considered to be fully responders to treatment (mean score ≤3) starting from T2.

**Figure 2:**
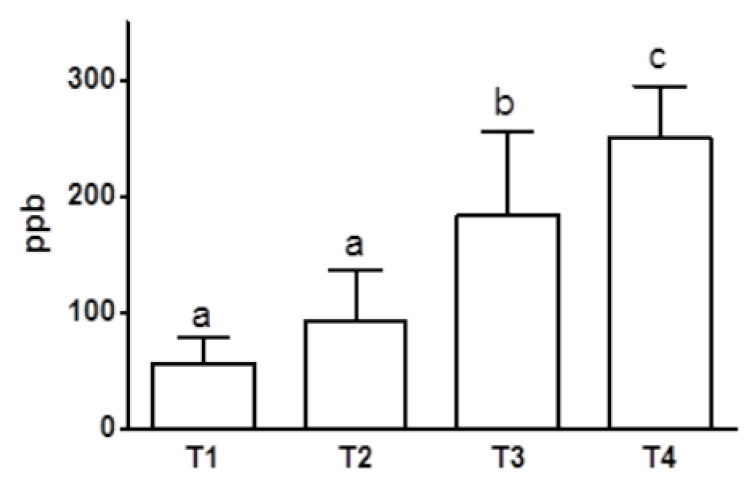
Blood fluoxetine concentrations after one (T1), two (T2), four (T3), and six (T4) months of fluoxetine treatment (1.5 mg/kg/day PO) (T1/T2 vs. T3/T4 *p* < 0.001; T3 vs. T4 *p* < 0.01; *n* = 8). a, b, c: statistically significant differences are present among the values reported in the bar of the graph.

Increasing concentrations of circulating fluoxetine and norfluoxetine were measured throughout the follow-up. Nevertheless, statistically significant (*p* < 0.01) increases in fluoxetine concentrations were observed starting from T3 with respect to T1–T2 values (Figure 2).

**Figure 3:**
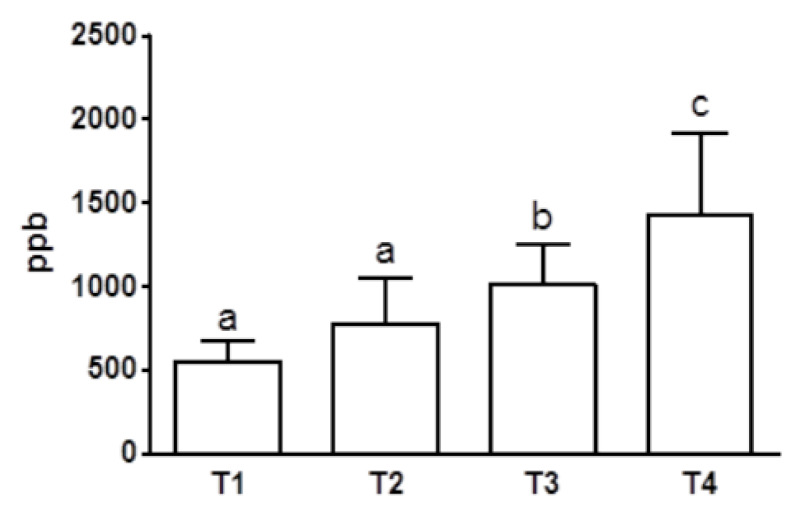
Blood norfluoxetine concentrations after one (T1), two (T2), four (T3), and six (T4) months of fluoxetine treatment (1.5 mg/kg/day PO) (T1/T2 vs. T3/T4 *p* < 0.01; *n* = 8). a, b, c: statistically significant differences are present among the values reported in the bar of the graph.

Accordingly, norfluoxetine levels increased significantly at T3 and T4 compared with those measured at previous time points (Figure 3).

According to Pearson’s correlation coefficient values at T4, a correlation between norfluoxetine levels and clinical scores (*r* = 0.75, *p* < 0.05) was observed.

**Figure 4:**
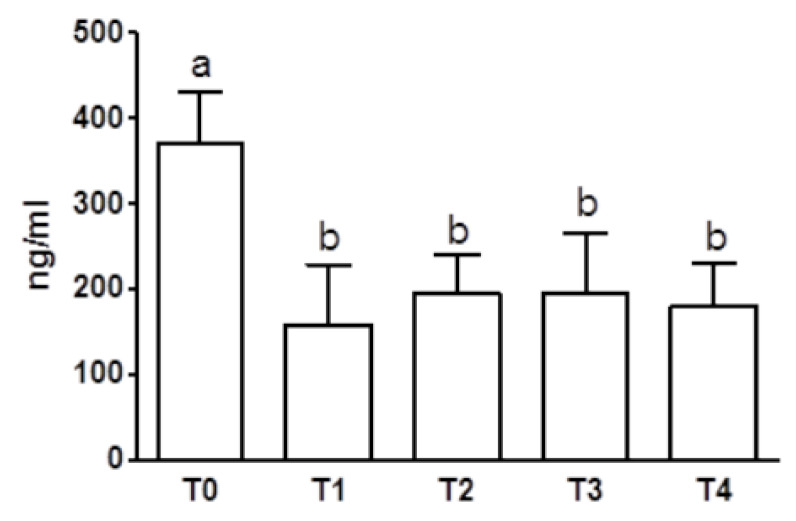
Blood 5-HT levels (mean values ± SD) in aggressive dogs (*n* = 8) at T0 (before treatment) and after one (T1), two (T2), four (T3), and six (T4) months of fluoxetine treatment (T0 vs. T1–T4, *p* < 0.001). a, b, c: statistically significant differences are present among the values reported in the bar of the graph.

Regarding blood 5-HT levels, a statistically significant decrease was observed between T0 and T1–T4 (*p* < 0.001) (Figure 4).

## 4. Discussion

Clinical outcomes observed in the present study seem to confirm those previously observed by Rosado et al. [11], although under different experimental conditions, suggesting a beneficial effect of fluoxetine for the treatment of canine aggression. In this study, the clinical response was evaluated at only one time point, namely after 30 days of treatment at 1 mg/kg, PO every 24 h. By contrast, very few data are available regarding effectiveness for periods for longer than six months of therapy. A dosage of 0.52 mg/kg/die has been considered effective for long-term treatment of dog aggression directed toward family members or strangers [13]. According to Duxbury [14], the appropriate fluoxetine dosage for the same behavioral problem would be 1 mg/kg/die. Thus, the recommended dosage range for fluoxetine in dogs varies from 0.5 to 2 mg/kg/die [15]. The dosage used in the present study (1.5 mg/kg/die) appeared effective in controlling aggression and was safe, as no signs of drug toxicity were recorded.

In our study, a significant improvement of clinical signs of aggression was observed after 1 month of therapy, although a full therapeutic effect was seen only at T2. The result is in line with what has been observed in previous studies reporting clinical improvement starting from 4 to 6 weeks after commencing treatment and can probably be ascribed to the pharmacokinetic and pharmacodynamic fluoxetine profile. In fact, due its long half-life and to the time required to induce changes at serotonergic receptor levels, fluoxetine has to be administered at least 6 to 8 weeks before evaluating clinical efficacy [16].

As already observed in human patients [17], in dogs too a great individual variability in plasma concentration of fluoxetine and norfluoxetine in response to a fixed dose seems to exist. From this point of view, in everyday veterinary practice, it should be considered that among factors influencing blood concentrations, besides individual variations in metabolic clearance rates, there is owner compliance in administering drug according to recommendations.

Increasing concentrations of fluoxetine and norfluoxetine were observed over time. There is little in literature describing fluoxetine pharmacokinetics, especially in dogs [18]. Nevertheless, this finding was expected as fluoxetine and its active metabolite exhibit a relatively slow elimination, especially in cases of chronic administration, leading to accumulation and delayed attainment of a steady state, even when a fixed dose is used.

While there was no evidence of a relationship between plasma fluoxetine concentrations and clinical response, Pearson’s correlation coefficient between circulating norfluoxetine levels and behavioral score improvement was acceptable, at least at T4. To the best of our knowledge, the correlation between plasma drug levels and therapeutic outcomes in the canine patient has never been investigated. Nevertheless, from a clinical point of view, this information could be important both to predict adequate or inadequate responses to treatment dose and to eventually establish the threshold blood concentration preserving efficacy while minimizing side effects. In human medicine, different studies investigating the possible relationships between plasma fluoxetine levels and therapeutic outcomes have led to conflicting results [17]. Our results seem to suggest that in dogs, the improvement of clinical scores is correlated with circulating levels of the active metabolite norfluoxetine rather than fluoxetine. Nevertheless, the correlation does not appear to be an early indicator of clinical efficacy.

Circulating 5-HT levels were decreased by treatment. The finding is in agreement with the results of previous studies on depression showing a significant decrease in platelet and plasma/serum 5-HT content following SSRIs administration [19,20] and with those by Rosado and colleagues [11]. According to the same authors, the modification in drug-circulating levels could be the result of a regulation phenomenon of peripheral 5-HT turnover after blocking the platelet 5-HT uptake place by fluoxetine. As in our study, all the dogs could be clinically classified as responders to treatment, so it might be suggested that blood 5-HT levels represent predicting markers of therapeutic outcomes. However, a great individual variability of neurotransmitter circulating levels was observed. It should also be considered that post-treatment serotonin concentrations seem to be conditioned by pre-treatment levels [11]. Further studies including a larger number of animals must be carried out before drawing conclusions about the prognostic significance of 5-HT peripheral levels in aggressive dogs treated with fluoxetine.

Despite the interesting findings, the study has some limitations. First, there was no untreated/placebo-treated group, due to ethical and practical reasons. Moreover, statistical results could have been influenced by the limited number of animals. However, the use of rigorous inclusion criteria allowed us to identify a homogeneous group of dogs with a precise diagnosis.

## 5. Conclusions

Overall, our data suggest that fluoxetine at the dose of 1.5 mg/kg/day associated with behavior treatment is effective and safe for long-term control of canine aggression directed toward owners. There is a poor correlation between plasma fluoxetine concentrations and clinical outcomes, whereas levels of norfluoxetine, the active metabolite, seem more reliable in predicting clinical efficacy. Moreover, fluoxetine therapy led to a decrease in circulating serotonin associated with clinical improvement. However, further research should be addressed to investigate whether this modification is predictive of successful therapeutic outcomes.

## Figures and Tables

**Table 1:** Evaluation of dog aggression on a five-point frequency scale (0–4).

**FREQUENCY**	
4	very high (at least 8 episodes/week)
3	high (5–7 episodes/week)
2	mild (2–4 episodes/week)
1	1 low (1 episode/week)
0	None
**INTENSITY**	
4	very high (aggression in absence of threat and without stop)
3	high (aggression in absence of threat and with stop)
2	mild (aggression in presence of threat and with stop)
1	low (aggression in form of a threat)
0	none
**DISTANCE**	
4	very high (within the social space of the proxemic sphere)
3	high (within the individual space of the proxemic sphere)
2	mild (following an attempt of physical contact)
1	low (following physical contact)
0	none
